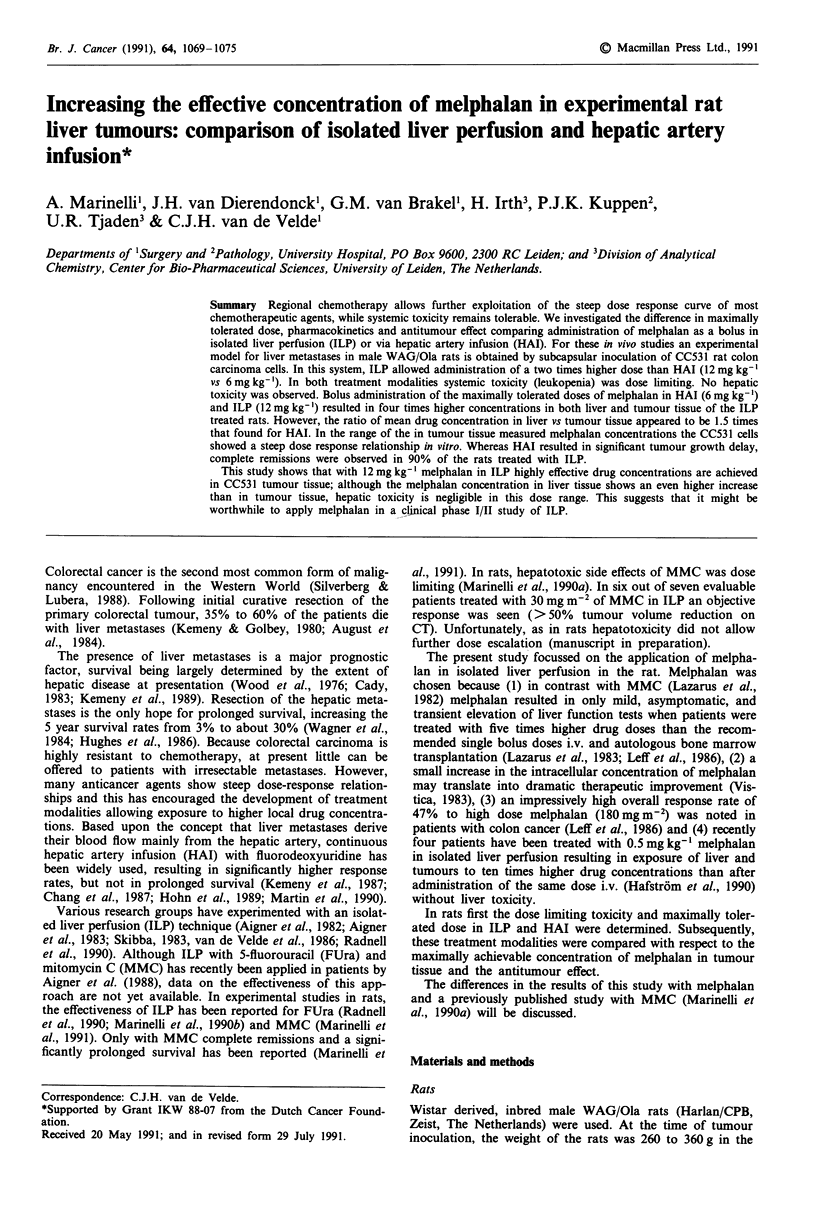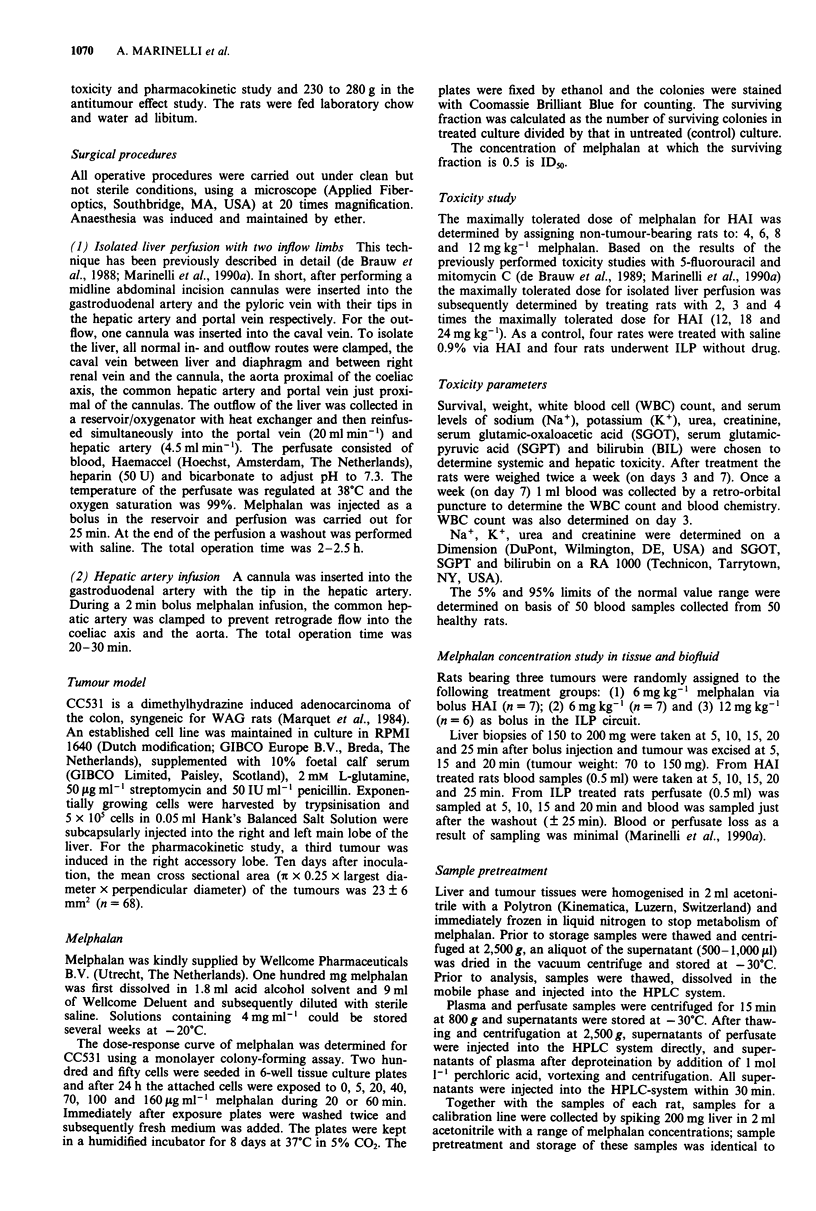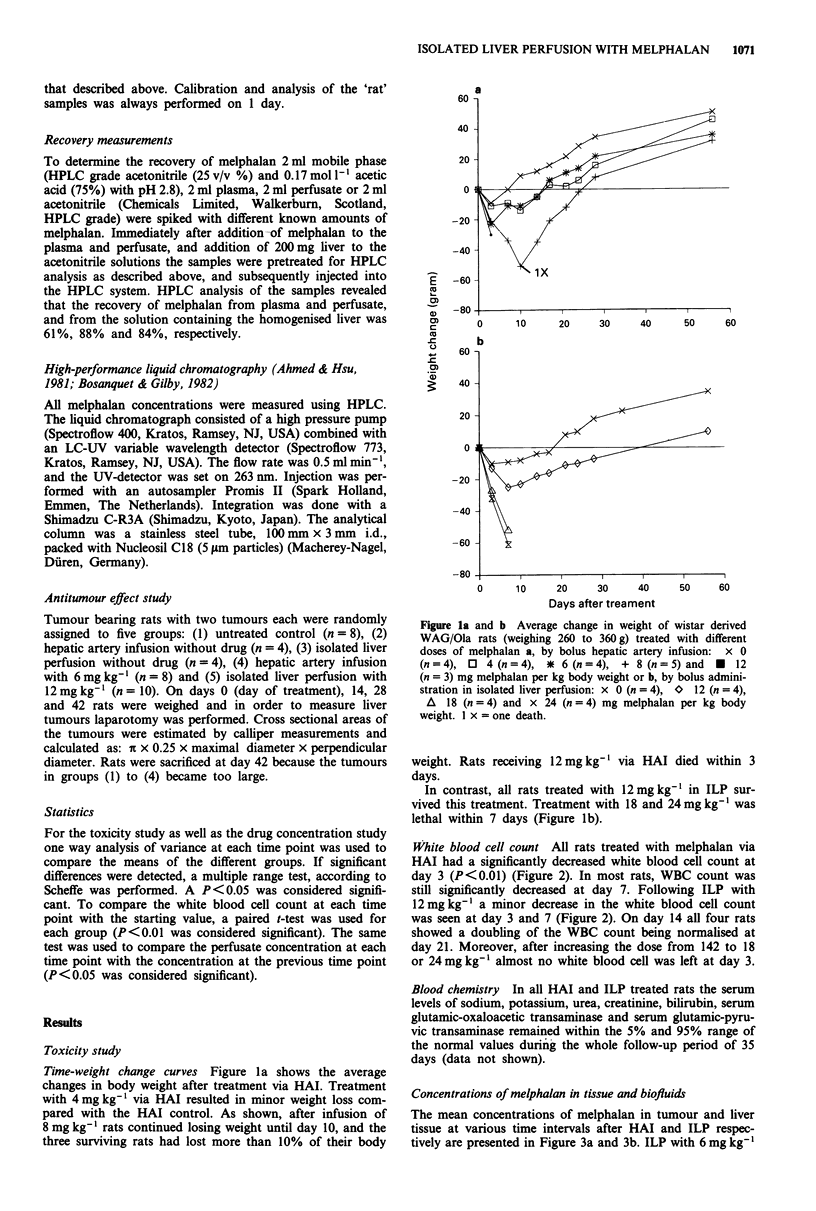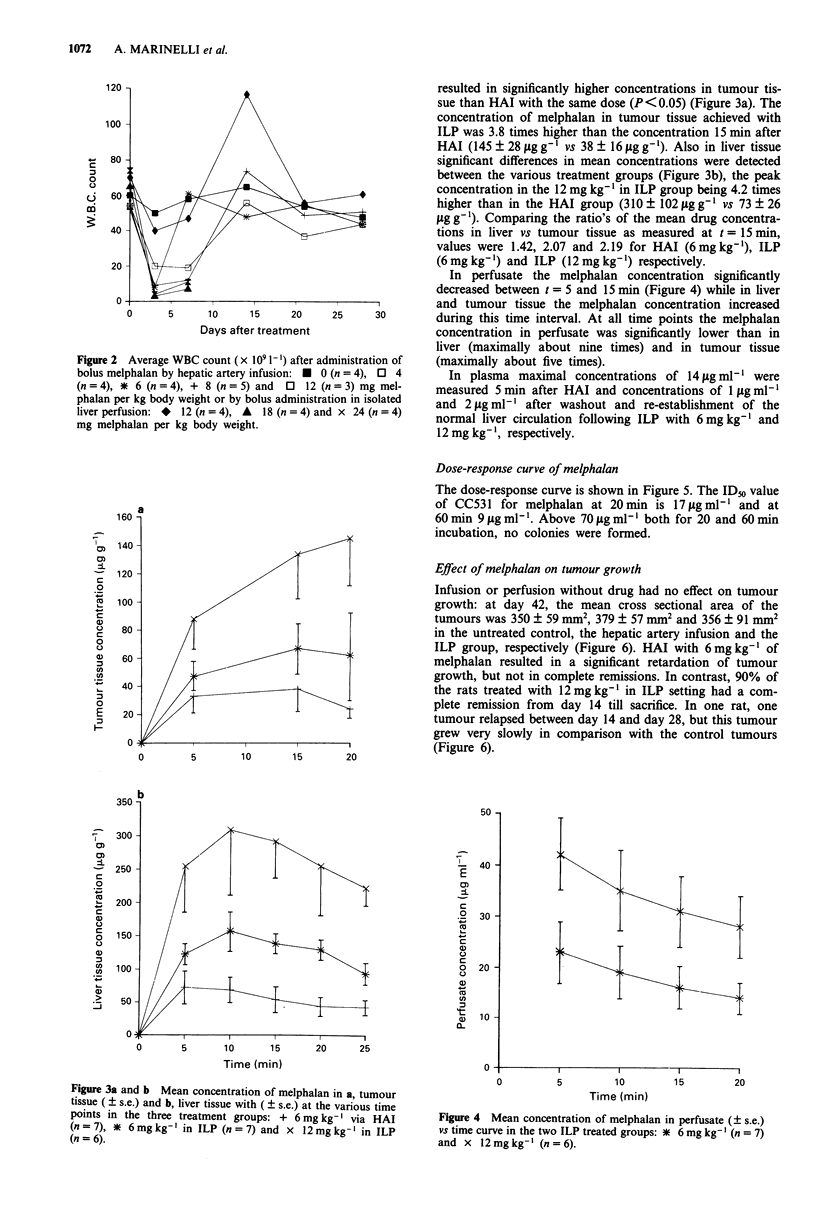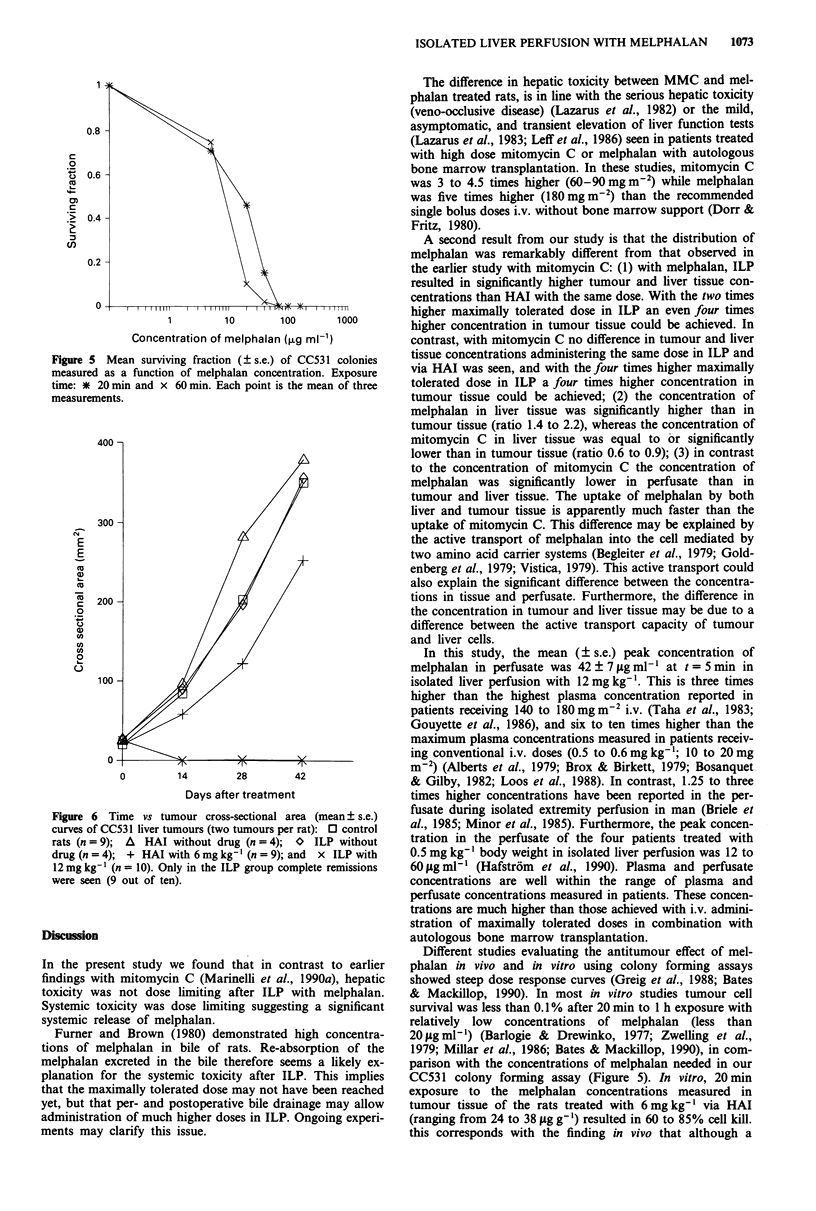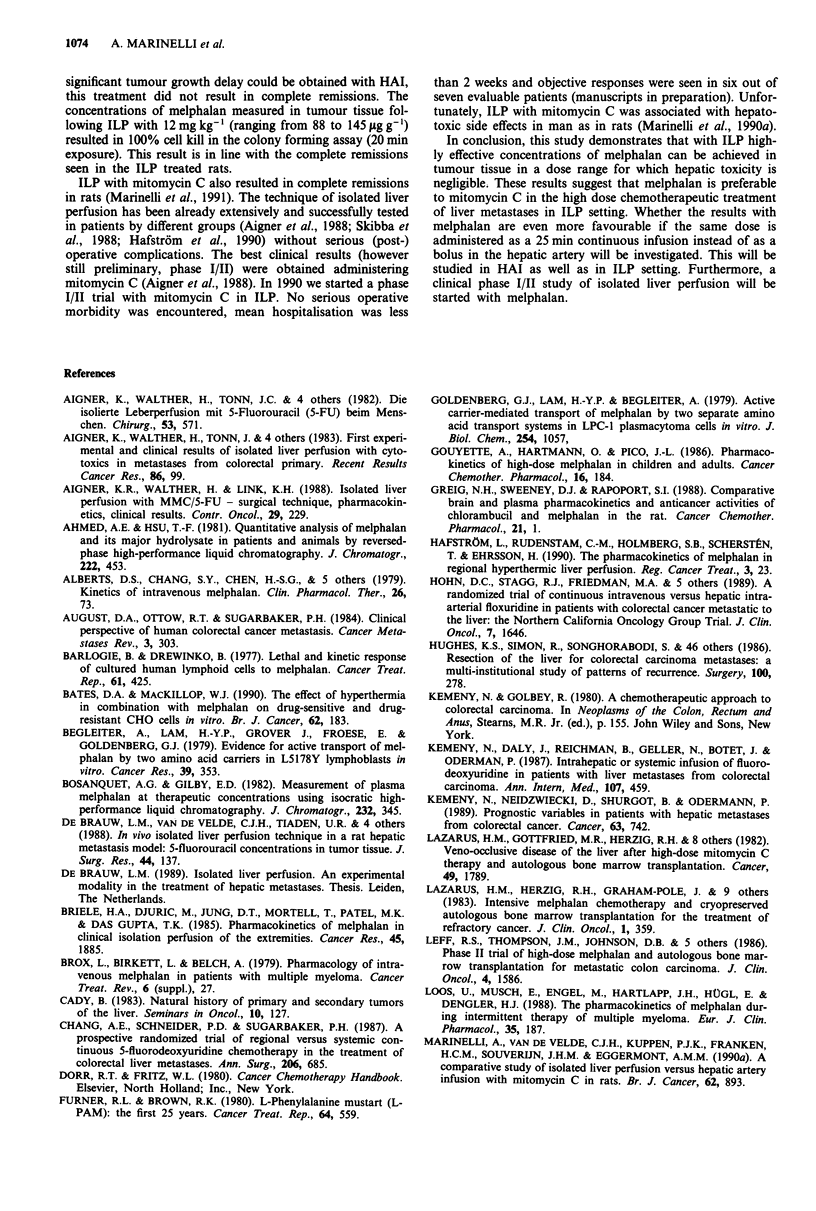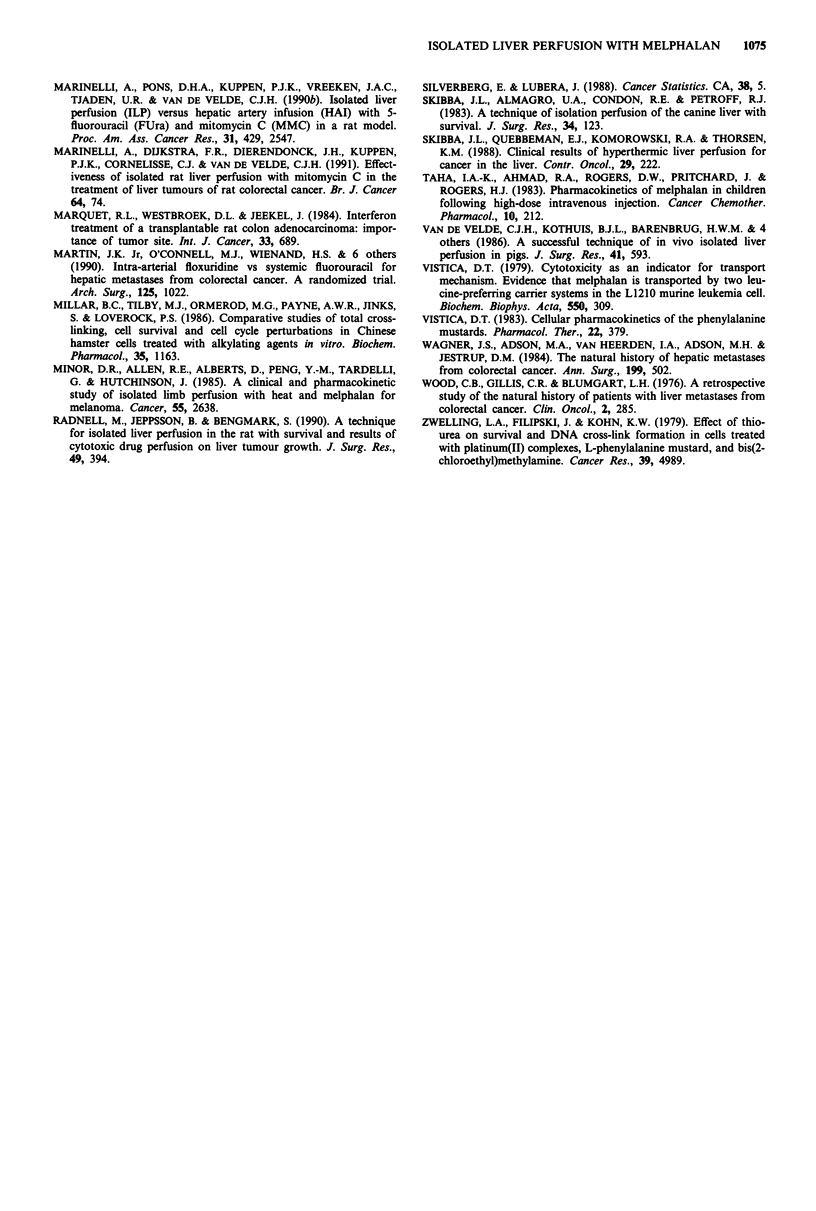# Increasing the effective concentration of melphalan in experimental rat liver tumours: comparison of isolated liver perfusion and hepatic artery infusion.

**DOI:** 10.1038/bjc.1991.466

**Published:** 1991-12

**Authors:** A. Marinelli, J. H. van Dierendonck, G. M. van Brakel, H. Irth, P. J. Kuppen, U. R. Tjaden, C. J. van de Velde

**Affiliations:** Departments of Surgery, University Hospital, Leiden, The Netherlands.

## Abstract

Regional chemotherapy allows further exploitation of the steep dose response curve of most chemotherapeutic agents, while systemic toxicity remains tolerable. We investigated the difference in maximally tolerated dose, pharmacokinetics and antitumour effect comparing administration of melphalan as a bolus in isolated liver perfusion (ILP) or via hepatic artery infusion (HAI). For these in vivo studies an experimental model for liver metastases in male WAG/Ola rats is obtained by subcapsular inoculation of CC531 rat colon carcinoma cells. In this system, ILP allowed administration of a two times higher dose than HAI (12 mg kg-1 vs 6 mg kg-1). In both treatment modalities systemic toxicity (leukopenia) was dose limiting. No hepatic toxicity was observed. Bolus administration of the maximally tolerated doses of melphalan in HAI (6 mg kg-1) and ILP (12 mg kg-1) resulted in four times higher concentrations in both liver and tumour tissue of the ILP treated rats. However, the ratio of mean drug concentration in liver vs tumour tissue appeared to be 1.5 times that found for HAI. In the range of the in tumour tissue measured melphalan concentrations the CC531 cells showed a steep dose response relationship in vitro. Whereas HAI resulted in significant tumour growth delay, complete remissions were observed in 90% of the rats treated with ILP. This study shows that with 12 mg kg-1 melphalan in ILP highly effective drug concentrations are achieved in CC531 tumour tissue; although the melphalan concentration in liver tissue shows an even higher increase than in tumour tissue, hepatic toxicity is negligible in this dose range.(ABSTRACT TRUNCATED AT 250 WORDS)


*Br. J. Cancer* (1991), **64**, 1069–1075 © Macmillan Press Ltd., 1991

### Increasing the effective concentration of melphalan in experimental rat liver tumours: comparison of isolated liver perfusion and hepatic artery infusion*

A. Marinelli[1], J.H. van Dierendonck[1], G.M. van Brakel[1], H. Irth[3], P.J.K. Kuppen[2], U.R. Tjaden[3] & C.J.H. van de Velde[1]

*Departments of [1]Surgery and [2]Pathology, University Hospital, PO Box 9600, 2300 RC Leiden; and [3]Division of Analytical Chemistry, Center for Bio-Pharmaceutical Sciences, University of Leiden, The Netherlands.*

**Summary** Regional chemotherapy allows further exploitation of the steep dose response curve of most chemotherapeutic agents, while systemic toxicity remains tolerable. We investigated the difference in maximally tolerated dose, pharmacokinetics and antitumour effect comparing administration of melphalan as a bolus in isolated liver perfusion (ILP) or via hepatic artery infusion (HAI). For these *in vivo* studies an experimental model for liver metastases in male WAG/Ola rats is obtained by subcapsular inoculation of CC531 rat colon carcinoma cells. In this system, ILP allowed administration of a two times higher dose than HAI (12 mg $kg^{-1}$ *vs* 6 mg $kg^{-1}$). In both treatment modalities systemic toxicity (leukopenia) was dose limiting. No hepatic toxicity was observed. Bolus administration of the maximally tolerated doses of melphalan in HAI (6 mg $kg^{-1}$) and ILP (12 mg $kg^{-1}$) resulted in four times higher concentrations in both liver and tumour tissue of the ILP treated rats. However, the ratio of mean drug concentration in liver *vs* tumour tissue appeared to be 1.5 times that found for HAI. In the range of the in tumour tissue measured melphalan concentrations the CC531 cells showed a steep dose response relationship *in vitro*. Whereas HAI resulted in significant tumour growth delay, complete remissions were observed in 90% of the rats treated with ILP.

This study shows that with 12 mg $kg^{-1}$ melphalan in ILP highly effective drug concentrations are achieved in CC531 tumour tissue; although the melphalan concentration in liver tissue shows an even higher increase than in tumour tissue, hepatic toxicity is negligible in this dose range. This suggests that it might be worthwhile to apply melphalan in a clinical phase I/II study of ILP.

Colorectal cancer is the second most common form of malignancy encountered in the Western World (Silverberg & Lubera, 1988). Following initial curative resection of the primary colorectal tumour, 35% to 60% of the patients die with liver metastases (Kemeny & Golbey, 1980; August *et al.*, 1984).

The presence of liver metastases is a major prognostic factor, survival being largely determined by the extent of hepatic disease at presentation (Wood *et al.*, 1976; Cady, 1983; Kemeny *et al.*, 1989). Resection of the hepatic metastases is the only hope for prolonged survival, increasing the 5 year survival rates from 3% to about 30% (Wagner *et al.*, 1984; Hughes *et al.*, 1986). Because colorectal carcinoma is highly resistant to chemotherapy, at present little can be offered to patients with irresectable metastases. However, many anticancer agents show steep dose-response relationships and this has encouraged the development of treatment modalities allowing exposure to higher local drug concentrations. Based upon the concept that liver metastases derive their blood flow mainly from the hepatic artery, continuous hepatic artery infusion (HAI) with fluorodeoxyuridine has been widely used, resulting in significantly higher response rates, but not in prolonged survival (Kemeny *et al.*, 1987; Chang *et al.*, 1987; Hohn *et al.*, 1989; Martin *et al.*, 1990).

Various research groups have experimented with an isolated liver perfusion (ILP) technique (Aigner *et al.*, 1982; Aigner *et al.*, 1983; Skibba, 1983, van de Velde *et al.*, 1986; Radnell *et al.*, 1990). Although ILP with 5-fluorouracil (FUra) and mitomycin C (MMC) has recently been applied in patients by Aigner *et al.* (1988), data on the effectiveness of this approach are not yet available. In experimental studies in rats, the effectiveness of ILP has been reported for FUra (Radnell *et al.*, 1990; Marinelli *et al.*, 1990*b*) and MMC (Marinelli *et al.*, 1991). Only with MMC complete remissions and a significantly prolonged survival has been reported (Marinelli *et al.*, 1991). In rats, hepatotoxic side effects of MMC was dose limiting (Marinelli *et al.*, 1990*a*). In six out of seven evaluable patients treated with 30 mg $m^{-2}$ of MMC in ILP an objective response was seen (>50% tumour volume reduction on CT). Unfortunately, as in rats hepatotoxicity did not allow further dose escalation (manuscript in preparation).

The present study focussed on the application of melphalan in isolated liver perfusion in the rat. Melphalan was chosen because (1) in contrast with MMC (Lazarus *et al.*, 1982) melphalan resulted in only mild, asymptomatic, and transient elevation of liver function tests when patients were treated with five times higher drug doses than the recommended single bolus doses i.v. and autologous bone marrow transplantation (Lazarus *et al.*, 1983; Leff *et al.*, 1986), (2) a small increase in the intracellular concentration of melphalan may translate into dramatic therapeutic improvement (Vistica, 1983), (3) an impressively high overall response rate of 47% to high dose melphalan (180 mg $m^{-2}$) was noted in patients with colon cancer (Leff *et al.*, 1986) and (4) recently four patients have been treated with 0.5 mg $kg^{-1}$ melphalan in isolated liver perfusion resulting in exposure of liver and tumours to ten times higher drug concentrations than after administration of the same dose i.v. (Hafström *et al.*, 1990) without liver toxicity.

In rats first the dose limiting toxicity and maximally tolerated dose in ILP and HAI were determined. Subsequently, these treatment modalities were compared with respect to the maximally achievable concentration of melphalan in tumour tissue and the antitumour effect.

The differences in the results of this study with melphalan and a previously published study with MMC (Marinelli *et al.*, 1990*a*) will be discussed.

### Materials and methods

### *Rats*

Wistar derived, inbred male WAG/Ola rats (Harlan/CPB, Zeist, The Netherlands) were used. At the time of tumour inoculation, the weight of the rats was 260 to 360 g in the

Correspondence: C.J.H. van de Velde.
*Supported by Grant IKW 88-07 from the Dutch Cancer Foundation.
Received 20 May 1991; and in revised form 29 July 1991.

toxicity and pharmacokinetic study and 230 to 280 g in the antitumour effect study. The rats were fed laboratory chow and water ad libitum.

*Surgical procedures*

All operative procedures were carried out under clean but not sterile conditions, using a microscope (Applied Fiberoptics, Southbridge, MA, USA) at 20 times magnification. Anaesthesia was induced and maintained by ether.

*(1) Isolated liver perfusion with two inflow limbs* This technique has been previously described in detail (de Brauw et al., 1988; Marinelli et al., 1990a). In short, after performing a midline abdominal incision cannulas were inserted into the gastroduodenal artery and the pyloric vein with their tips in the hepatic artery and portal vein respectively. For the outflow, one cannula was inserted into the caval vein. To isolate the liver, all normal in- and outflow routes were clamped, the caval vein between liver and diaphragm and between right renal vein and the cannula, the aorta proximal of the coeliac axis, the common hepatic artery and portal vein just proximal of the cannulas. The outflow of the liver was collected in a reservoir/oxygenator with heat exchanger and then reinfused simultaneously into the portal vein (20 ml $min^{-1}$) and hepatic artery (4.5 ml $min^{-1}$). The perfusate consisted of blood, Haemaccel (Hoechst, Amsterdam, The Netherlands), heparin (50 U) and bicarbonate to adjust pH to 7.3. The temperature of the perfusate was regulated at 38°C and the oxygen saturation was 99%. Melphalan was injected as a bolus in the reservoir and perfusion was carried out for 25 min. At the end of the perfusion a washout was performed with saline. The total operation time was 2-2.5 h.

*(2) Hepatic artery infusion* A cannula was inserted into the gastroduodenal artery with the tip in the hepatic artery. During a 2 min bolus melphalan infusion, the common hepatic artery was clamped to prevent retrograde flow into the coeliac axis and the aorta. The total operation time was 20-30 min.

*Tumour model*

CC531 is a dimethylhydrazine induced adenocarcinoma of the colon, syngeneic for WAG rats (Marquet et al., 1984). An established cell line was maintained in culture in RPMI 1640 (Dutch modification; GIBCO Europe B.V., Breda, The Netherlands), supplemented with 10% foetal calf serum (GIBCO Limited, Paisley, Scotland), 2 mM L-glutamine, 50 $\mu$g $ml^{-1}$ streptomycin and 50 IU $ml^{-1}$ penicillin. Exponentially growing cells were harvested by trypsinisation and $5 \times 10^5$ cells in 0.05 ml Hank's Balanced Salt Solution were subcapsularly injected into the right and left main lobe of the liver. For the pharmacokinetic study, a third tumour was induced in the right accessory lobe. Ten days after inoculation, the mean cross sectional area ($\pi \times 0.25 \times$ largest diameter $\times$ perpendicular diameter) of the tumours was 23 ± 6 $mm^2$ ($n = 68$).

*Melphalan*

Melphalan was kindly supplied by Wellcome Pharmaceuticals B.V. (Utrecht, The Netherlands). One hundred mg melphalan was first dissolved in 1.8 ml acid alcohol solvent and 9 ml of Wellcome Deluent and subsequently diluted with sterile saline. Solutions containing 4 mg $ml^{-1}$ could be stored several weeks at −20°C.

The dose-response curve of melphalan was determined for CC531 using a monolayer colony-forming assay. Two hundred and fifty cells were seeded in 6-well tissue culture plates and after 24 h the attached cells were exposed to 0, 5, 20, 40, 70, 100 and 160 $\mu$g $ml^{-1}$ melphalan during 20 or 60 min. Immediately after exposure plates were washed twice and subsequently fresh medium was added. The plates were kept in a humidified incubator for 8 days at 37°C in 5% $CO_2$. The plates were fixed by ethanol and the colonies were stained with Coomassie Brilliant Blue for counting. The surviving fraction was calculated as the number of surviving colonies in treated culture divided by that in untreated (control) culture.

The concentration of melphalan at which the surviving fraction is 0.5 is $ID_{50}$.

*Toxicity study*

The maximally tolerated dose of melphalan for HAI was determined by assigning non-tumour-bearing rats to: 4, 6, 8 and 12 mg $kg^{-1}$ melphalan. Based on the results of the previously performed toxicity studies with 5-fluorouracil and mitomycin C (de Brauw et al., 1989; Marinelli et al., 1990a) the maximally tolerated dose for isolated liver perfusion was subsequently determined by treating rats with 2, 3 and 4 times the maximally tolerated dose for HAI (12, 18 and 24 mg $kg^{-1}$). As a control, four rates were treated with saline 0.9% via HAI and four rats underwent ILP without drug.

*Toxicity parameters*

Survival, weight, white blood cell (WBC) count, and serum levels of sodium ($Na^+$), potassium ($K^+$), urea, creatinine, serum glutamic-oxaloacetic acid (SGOT), serum glutamic-pyruvic acid (SGPT) and bilirubin (BIL) were chosen to determine systemic and hepatic toxicity. After treatment the rats were weighed twice a week (on days 3 and 7). Once a week (on day 7) 1 ml blood was collected by a retro-orbital puncture to determine the WBC count and blood chemistry. WBC count was also determined on day 3.

$Na^+$, $K^+$, urea and creatinine were determined on a Dimension (DuPont, Wilmington, DE, USA) and SGOT, SGPT and bilirubin on a RA 1000 (Technicon, Tarrytown, NY, USA).

The 5% and 95% limits of the normal value range were determined on basis of 50 blood samples collected from 50 healthy rats.

*Melphalan concentration study in tissue and biofluid*

Rats bearing three tumours were randomly assigned to the following treatment groups: (1) 6 mg $kg^{-1}$ melphalan via bolus HAI ($n = 7$); (2) 6 mg $kg^{-1}$ ($n = 7$) and (3) 12 mg $kg^{-1}$ ($n = 6$) as bolus in the ILP circuit.

Liver biopsies of 150 to 200 mg were taken at 5, 10, 15, 20 and 25 min after bolus injection and tumour was excised at 5, 15 and 20 min (tumour weight: 70 to 150 mg). From HAI treated rats blood samples (0.5 ml) were taken at 5, 10, 15, 20 and 25 min. From ILP treated rats perfusate (0.5 ml) was sampled at 5, 10, 15 and 20 min and blood was sampled just after the washout (± 25 min). Blood or perfusate loss as a result of sampling was minimal (Marinelli et al., 1990a).

*Sample pretreatment*

Liver and tumour tissues were homogenised in 2 ml acetonitrile with a Polytron (Kinematica, Luzern, Switzerland) and immediately frozen in liquid nitrogen to stop metabolism of melphalan. Prior to storage samples were thawed and centrifuged at 2,500 g, an aliquot of the supernatant (500-1,000 $\mu$l) was dried in the vacuum centrifuge and stored at −30°C. Prior to analysis, samples were thawed, dissolved in the mobile phase and injected into the HPLC system.

Plasma and perfusate samples were centrifuged for 15 min at 800 g and supernatants were stored at −30°C. After thawing and centrifugation at 2,500 g, supernatants of perfusate were injected into the HPLC system directly, and supernatants of plasma after deproteination by addition of 1 mol $l^{-1}$ perchloric acid, vortexing and centrifugation. All supernatants were injected into the HPLC-system within 30 min.

Together with the samples of each rat, samples for a calibration line were collected by spiking 200 mg liver in 2 ml acetonitrile with a range of melphalan concentrations; sample pretreatment and storage of these samples was identical to that described above. Calibration and analysis of the 'rat' samples was always performed on 1 day.

*Recovery measurements*

To determine the recovery of melphalan 2 ml mobile phase (HPLC grade acetonitrile (25 v/v %) and 0.17 mol $l^{-1}$ acetic acid (75%) with pH 2.8), 2 ml plasma, 2 ml perfusate or 2 ml acetonitrile (Chemicals Limited, Walkerburn, Scotland, HPLC grade) were spiked with different known amounts of melphalan. Immediately after addition of melphalan to the plasma and perfusate, and addition of 200 mg liver to the acetonitrile solutions the samples were pretreated for HPLC analysis as described above, and subsequently injected into the HPLC system. HPLC analysis of the samples revealed that the recovery of melphalan from plasma and perfusate, and from the solution containing the homogenised liver was 61%, 88% and 84%, respectively.

*High-performance liquid chromatography (Ahmed & Hsu, 1981; Bosanquet & Gilby, 1982)*

All melphalan concentrations were measured using HPLC. The liquid chromatograph consisted of a high pressure pump (Spectroflow 400, Kratos, Ramsey, NJ, USA) combined with an LC-UV variable wavelength detector (Spectroflow 773, Kratos, Ramsey, NJ, USA). The flow rate was 0.5 ml $min^{-1}$, and the UV-detector was set on 263 nm. Injection was performed with an autosampler Promis II (Spark Holland, Emmen, The Netherlands). Integration was done with a Shimadzu C-R3A (Shimadzu, Kyoto, Japan). The analytical column was a stainless steel tube, 100 mm × 3 mm i.d., packed with Nucleosil C18 (5 μm particles) (Macherey-Nagel, Düren, Germany).

*Antitumour effect study*

Tumour bearing rats with two tumours each were randomly assigned to five groups: (1) untreated control ($n = 8$), (2) hepatic artery infusion without drug ($n = 4$), (3) isolated liver perfusion without drug ($n = 4$), (4) hepatic artery infusion with 6 mg $kg^{-1}$ ($n = 8$) and (5) isolated liver perfusion with 12 mg $kg^{-1}$ ($n = 10$). On days 0 (day of treatment), 14, 28 and 42 rats were weighed and in order to measure liver tumours laparotomy was performed. Cross sectional areas of the tumours were estimated by calliper measurements and calculated as: $\pi \times 0.25 \times$ maximal diameter × perpendicular diameter. Rats were sacrificed at day 42 because the tumours in groups (1) to (4) became too large.

*Statistics*

For the toxicity study as well as the drug concentration study one way analysis of variance at each time point was used to compare the means of the different groups. If significant differences were detected, a multiple range test, according to Scheffe was performed. A $P < 0.05$ was considered significant. To compare the white blood cell count at each time point with the starting value, a paired $t$-test was used for each group ($P < 0.01$ was considered significant). The same test was used to compare the perfusate concentration at each time point with the concentration at the previous time point ($P < 0.05$ was considered significant).

### Results

*Toxicity study*

*Time-weight change curves* Figure 1a shows the average changes in body weight after treatment via HAI. Treatment with 4 mg $kg^{-1}$ via HAI resulted in minor weight loss compared with the HAI control. As shown, after infusion of 8 mg $kg^{-1}$ rats continued losing weight until day 10, and the three surviving rats had lost more than 10% of their body

Figure 1a and b Average change in weight of wistar derived WAG/Ola rats (weighing 260 to 360 g) treated with different doses of melphalan **a**, by bolus hepatic artery infusion: × 0 ($n = 4$), □ 4 ($n = 4$), ✱ 6 ($n = 4$), + 8 ($n = 5$) and ■ 12 ($n = 3$) mg melphalan per kg body weight or **b**, by bolus administration in isolated liver perfusion: × 0 ($n = 4$), ◇ 12 ($n = 4$), △ 18 ($n = 4$) and × 24 ($n = 4$) mg melphalan per kg body weight. 1 × = one death.

weight. Rats receiving 12 mg $kg^{-1}$ via HAI died within 3 days.

In contrast, all rats treated with 12 mg $kg^{-1}$ in ILP survived this treatment. Treatment with 18 and 24 mg $kg^{-1}$ was lethal within 7 days (Figure 1b).

*White blood cell count* All rats treated with melphalan via HAI had a significantly decreased white blood cell count at day 3 ($P < 0.01$) (Figure 2). In most rats, WBC count was still significantly decreased at day 7. Following ILP with 12 mg $kg^{-1}$ a minor decrease in the white blood cell count was seen at day 3 and 7 (Figure 2). On day 14 all four rats showed a doubling of the WBC count being normalised at day 21. Moreover, after increasing the dose from 142 to 18 or 24 mg $kg^{-1}$ almost no white blood cell was left at day 3.

*Blood chemistry* In all HAI and ILP treated rats the serum levels of sodium, potassium, urea, creatinine, bilirubin, serum glutamic-oxaloacetic transaminase and serum glutamic-pyruvic transaminase remained within the 5% and 95% range of the normal values during the whole follow-up period of 35 days (data not shown).

*Concentrations of melphalan in tissue and biofluids*

The mean concentrations of melphalan in tumour and liver tissue at various time intervals after HAI and ILP respectively are presented in Figure 3a and 3b. ILP with 6 mg $kg^{-1}$

**Figure 2** Average WBC count ( × $10^9\,l^{-1}$) after administration of bolus melphalan by hepatic artery infusion: ■ 0 ($n = 4$), □ 4 ($n = 4$), ✱ 6 ($n = 4$), + 8 ($n = 5$) and □ 12 ($n = 3$) mg melphalan per kg body weight or by bolus administration in isolated liver perfusion: ◆ 12 ($n = 4$), ▲ 18 ($n = 4$) and × 24 ($n = 4$) mg melphalan per kg body weight.

**Figure 3a and b** Mean concentration of melphalan in **a**, tumour tissue ( ± s.e.) and **b**, liver tissue with ( ± s.e.) at the various time points in the three treatment groups: + 6 mg $kg^{-1}$ via HAI ($n = 7$), ✱ 6 mg $kg^{-1}$ in ILP ($n = 7$) and × 12 mg $kg^{-1}$ in ILP ($n = 6$).

resulted in significantly higher concentrations in tumour tissue than HAI with the same dose ($P < 0.05$) (Figure 3a). The concentration of melphalan in tumour tissue achieved with ILP was 3.8 times higher than the concentration 15 min after HAI (145 ± 28 μg $g^{-1}$ *vs* 38 ± 16 μg $g^{-1}$). Also in liver tissue significant differences in mean concentrations were detected between the various treatment groups (Figure 3b), the peak concentration in the 12 mg $kg^{-1}$ in ILP group being 4.2 times higher than in the HAI group (310 ± 102 μg $g^{-1}$ *vs* 73 ± 26 μg $g^{-1}$). Comparing the ratio's of the mean drug concentrations in liver *vs* tumour tissue as measured at $t = 15$ min, values were 1.42, 2.07 and 2.19 for HAI (6 mg $kg^{-1}$), ILP (6 mg $kg^{-1}$) and ILP (12 mg $kg^{-1}$) respectively.

In perfusate the melphalan concentration significantly decreased between $t = 5$ and 15 min (Figure 4) while in liver and tumour tissue the melphalan concentration increased during this time interval. At all time points the melphalan concentration in perfusate was significantly lower than in liver (maximally about nine times) and in tumour tissue (maximally about five times).

In plasma maximal concentrations of 14 μg $ml^{-1}$ were measured 5 min after HAI and concentrations of 1 μg $ml^{-1}$ and 2 μg $ml^{-1}$ after washout and re-establishment of the normal liver circulation following ILP with 6 mg $kg^{-1}$ and 12 mg $kg^{-1}$, respectively.

### *Dose-response curve of melphalan*

The dose-response curve is shown in Figure 5. The $ID_{50}$ value of CC531 for melphalan at 20 min is 17 μg $ml^{-1}$ and at 60 min 9 μg $ml^{-1}$. Above 70 μg $ml^{-1}$ both for 20 and 60 min incubation, no colonies were formed.

### *Effect of melphalan on tumour growth*

Infusion or perfusion without drug had no effect on tumour growth: at day 42, the mean cross sectional area of the tumours was 350 ± 59 $mm^2$, 379 ± 57 $mm^2$ and 356 ± 91 $mm^2$ in the untreated control, the hepatic artery infusion and the ILP group, respectively (Figure 6). HAI with 6 mg $kg^{-1}$ of melphalan resulted in a significant retardation of tumour growth, but not in complete remissions. In contrast, 90% of the rats treated with 12 mg $kg^{-1}$ in ILP setting had a complete remission from day 14 till sacrifice. In one rat, one tumour relapsed between day 14 and day 28, but this tumour grew very slowly in comparison with the control tumours (Figure 6).

**Figure 4** Mean concentration of melphalan in perfusate (± s.e.) *vs* time curve in the two ILP treated groups: ✱ 6 mg $kg^{-1}$ ($n = 7$) and × 12 mg $kg^{-1}$ ($n = 6$).

**Figure 5** Mean surviving fraction (± s.e.) of CC531 colonies measured as a function of melphalan concentration. Exposure time: ✱ 20 min and × 60 min. Each point is the mean of three measurements.

**Figure 6** Time *vs* tumour cross-sectional area (mean ± s.e.) curves of CC531 liver tumours (two tumours per rat): □ control rats ($n = 9$); △ HAI without drug ($n = 4$); ◇ ILP without drug ($n = 4$); + HAI with 6 mg kg$^{-1}$ ($n = 9$); and × ILP with 12 mg kg$^{-1}$ ($n = 10$). Only in the ILP group complete remissions were seen (9 out of ten).

### Discussion

In the present study we found that in contrast to earlier findings with mitomycin C (Marinelli *et al.*, 1990*a*), hepatic toxicity was not dose limiting after ILP with melphalan. Systemic toxicity was dose limiting suggesting a significant systemic release of melphalan.

Furner and Brown (1980) demonstrated high concentrations of melphalan in bile of rats. Re-absorption of the melphalan excreted in the bile therefore seems a likely explanation for the systemic toxicity after ILP. This implies that the maximally tolerated dose may not have been reached yet, but that per- and postoperative bile drainage may allow administration of much higher doses in ILP. Ongoing experiments may clarify this issue.

The difference in hepatic toxicity between MMC and melphalan treated rats, is in line with the serious hepatic toxicity (veno-occlusive disease) (Lazarus *et al.*, 1982) or the mild, asymptomatic, and transient elevation of liver function tests (Lazarus *et al.*, 1983; Leff *et al.*, 1986) seen in patients treated with high dose mitomycin C or melphalan with autologous bone marrow transplantation. In these studies, mitomycin C was 3 to 4.5 times higher (60–90 mg m$^{-2}$) while melphalan was five times higher (180 mg m$^{-2}$) than the recommended single bolus doses i.v. without bone marrow support (Dorr & Fritz, 1980).

A second result from our study is that the distribution of melphalan was remarkably different from that observed in the earlier study with mitomycin C: (1) with melphalan, ILP resulted in significantly higher tumour and liver tissue concentrations than HAI with the same dose. With the *two* times higher maximally tolerated dose in ILP an even *four* times higher concentration in tumour tissue could be achieved. In contrast, with mitomycin C no difference in tumour and liver tissue concentrations administering the same dose in ILP and via HAI was seen, and with the *four* times higher maximally tolerated dose in ILP a *four* times higher concentration in tumour tissue could be achieved; (2) the concentration of melphalan in liver tissue was significantly higher than in tumour tissue (ratio 1.4 to 2.2), whereas the concentration of mitomycin C in liver tissue was equal to or significantly lower than in tumour tissue (ratio 0.6 to 0.9); (3) in contrast to the concentration of mitomycin C the concentration of melphalan was significantly lower in perfusate than in tumour and liver tissue. The uptake of melphalan by both liver and tumour tissue is apparently much faster than the uptake of mitomycin C. This difference may be explained by the active transport of melphalan into the cell mediated by two amino acid carrier systems (Begleiter *et al.*, 1979; Goldenberg *et al.*, 1979; Vistica, 1979). This active transport could also explain the significant difference between the concentrations in tissue and perfusate. Furthermore, the difference in the concentration in tumour and liver tissue may be due to a difference between the active transport capacity of tumour and liver cells.

In this study, the mean (± s.e.) peak concentration of melphalan in perfusate was 42 ± 7 μg ml$^{-1}$ at $t = 5$ min in isolated liver perfusion with 12 mg kg$^{-1}$. This is three times higher than the highest plasma concentration reported in patients receiving 140 to 180 mg m$^{-2}$ i.v. (Taha *et al.*, 1983; Gouyette *et al.*, 1986), and six to ten times higher than the maximum plasma concentrations measured in patients receiving conventional i.v. doses (0.5 to 0.6 mg kg$^{-1}$; 10 to 20 mg m$^{-2}$) (Alberts *et al.*, 1979; Brox & Birkett, 1979; Bosanquet & Gilby, 1982; Loos *et al.*, 1988). In contrast, 1.25 to three times higher concentrations have been reported in the perfusate during isolated extremity perfusion in man (Briele *et al.*, 1985; Minor *et al.*, 1985). Furthermore, the peak concentration in the perfusate of the four patients treated with 0.5 mg kg$^{-1}$ body weight in isolated liver perfusion was 12 to 60 μg ml$^{-1}$ (Hafström *et al.*, 1990). Plasma and perfusate concentrations are well within the range of plasma and perfusate concentrations measured in patients. These concentrations are much higher than those achieved with i.v. administration of maximally tolerated doses in combination with autologous bone marrow transplantation.

Different studies evaluating the antitumour effect of melphalan *in vivo* and *in vitro* using colony forming assays showed steep dose response curves (Greig *et al.*, 1988; Bates & Mackillop, 1990). In most *in vitro* studies tumour cell survival was less than 0.1% after 20 min to 1 h exposure with relatively low concentrations of melphalan (less than 20 μg ml$^{-1}$) (Barlogie & Drewinko, 1977; Zwelling *et al.*, 1979; Millar *et al.*, 1986; Bates & Mackillop, 1990), in comparison with the concentrations of melphalan needed in our CC531 colony forming assay (Figure 5). *In vitro*, 20 min exposure to the melphalan concentrations measured in tumour tissue of the rats treated with 6 mg kg$^{-1}$ via HAI (ranging from 24 to 38 μg g$^{-1}$) resulted in 60 to 85% cell kill. this corresponds with the finding *in vivo* that although a significant tumour growth delay could be obtained with HAI, this treatment did not result in complete remissions. The concentrations of melphalan measured in tumour tissue following ILP with 12 mg $kg^{-1}$ (ranging from 88 to 145 μg $g^{-1}$) resulted in 100% cell kill in the colony forming assay (20 min exposure). This result is in line with the complete remissions seen in the ILP treated rats.

ILP with mitomycin C also resulted in complete remissions in rats (Marinelli et al., 1991). The technique of isolated liver perfusion has been already extensively and successfully tested in patients by different groups (Aigner et al., 1988; Skibba et al., 1988; Hafström et al., 1990) without serious (post-) operative complications. The best clinical results (however still preliminary, phase I/II) were obtained administering mitomycin C (Aigner et al., 1988). In 1990 we started a phase I/II trial with mitomycin C in ILP. No serious operative morbidity was encountered, mean hospitalisation was less than 2 weeks and objective responses were seen in six out of seven evaluable patients (manuscripts in preparation). Unfortunately, ILP with mitomycin C was associated with hepatotoxic side effects in man as in rats (Marinelli et al., 1990a).

In conclusion, this study demonstrates that with ILP highly effective concentrations of melphalan can be achieved in tumour tissue in a dose range for which hepatic toxicity is negligible. These results suggest that melphalan is preferable to mitomycin C in the high dose chemotherapeutic treatment of liver metastases in ILP setting. Whether the results with melphalan are even more favourable if the same dose is administered as a 25 min continuous infusion instead of as a bolus in the hepatic artery will be investigated. This will be studied in HAI as well as in ILP setting. Furthermore, a clinical phase I/II study of isolated liver perfusion will be started with melphalan.

### References

AIGNER, K., WALTHER, H., TONN, J.C. & 4 others (1982). Die isolierte Leberperfusion mit 5-Fluorouracil (5-FU) beim Menschen. *Chirurg.*, **53**, 571.

AIGNER, K., WALTHER, H., TONN, J. & 4 others (1983). First experimental and clinical results of isolated liver perfusion with cytotoxics in metastases from colorectal primary. *Recent Results Cancer Res.*, **86**, 99.

AIGNER, K.R., WALTHER, H. & LINK, K.H. (1988). Isolated liver perfusion with MMC/5-FU – surgical technique, pharmacokinetics, clinical results. *Contr. Oncol.*, **29**, 229.

AHMED, A.E. & HSU, T.-F. (1981). Quantitative analysis of melphalan and its major hydrolysate in patients and animals by reversed-phase high-performance liquid chromatography. *J. Chromatogr.*, **222**, 453.

ALBERTS, D.S., CHANG, S.Y., CHEN, H.-S.G., & 5 others (1979). Kinetics of intravenous melphalan. *Clin. Pharmacol. Ther.*, **26**, 73.

AUGUST, D.A., OTTOW, R.T. & SUGARBAKER, P.H. (1984). Clinical perspective of human colorectal cancer metastasis. *Cancer Metastases Rev.*, **3**, 303.

BARLOGIE, B. & DREWINKO, B. (1977). Lethal and kinetic response of cultured human lymphoid cells to melphalan. *Cancer Treat. Rep.*, **61**, 425.

BATES, D.A. & MACKILLOP, W.J. (1990). The effect of hyperthermia in combination with melphalan on drug-sensitive and drug-resistant CHO cells *in vitro*. *Br. J. Cancer*, **62**, 183.

BEGLEITER, A., LAM, H.-Y.P., GROVER J., FROESE, E. & GOLDENBERG, G.J. (1979). Evidence for active transport of melphalan by two amino acid carriers in L5178Y lymphoblasts *in vitro*. *Cancer Res.*, **39**, 353.

BOSANQUET, A.G. & GILBY, E.D. (1982). Measurement of plasma melphalan at therapeutic concentrations using isocratic high-performance liquid chromatography. *J. Chromatogr.*, **232**, 345.

DE BRAUW, L.M., VAN DE VELDE, C.J.H., TIADEN, U.R. & 4 others (1988). *In vivo* isolated liver perfusion technique in a rat hepatic metastasis model: 5-fluorouracil concentrations in tumor tissue. *J. Surg. Res.*, **44**, 137.

DE BRAUW, L.M. (1989). Isolated liver perfusion. An experimental modality in the treatment of hepatic metastases. Thesis. Leiden, The Netherlands.

BRIELE, H.A., DJURIC, M., JUNG, D.T., MORTELL, T., PATEL, M.K. & DAS GUPTA, T.K. (1985). Pharmacokinetics of melphalan in clinical isolation perfusion of the extremities. *Cancer Res.*, **45**, 1885.

BROX, L., BIRKETT, L. & BELCH, A. (1979). Pharmacology of intravenous melphalan in patients with multiple myeloma. *Cancer Treat. Rev.*, **6** (suppl.), 27.

CADY, B. (1983). Natural history of primary and secondary tumors of the liver. *Seminars in Oncol.*, **10**, 127.

CHANG, A.E., SCHNEIDER, P.D. & SUGARBAKER, P.H. (1987). A prospective randomized trial of regional versus systemic continuous 5-fluorodeoxyuridine chemotherapy in the treatment of colorectal liver metastases. *Ann. Surg.*, **206**, 685.

DORR, R.T. & FRITZ, W.L. (1980). *Cancer Chemotherapy Handbook*. Elsevier, North Holland; Inc., New York.

FURNER, R.L. & BROWN, R.K. (1980). L-Phenylalanine mustart (L-PAM): the first 25 years. *Cancer Treat. Rep.*, **64**, 559.

GOLDENBERG, G.J., LAM, H.-Y.P. & BEGLEITER, A. (1979). Active carrier-mediated transport of melphalan by two separate amino acid transport systems in LPC-1 plasmacytoma cells *in vitro*. *J. Biol. Chem.*, **254**, 1057.

GOUYETTE, A., HARTMANN, O. & PICO, J.-L. (1986). Pharmacokinetics of high-dose melphalan in children and adults. *Cancer Chemother. Pharmacol.*, **16**, 184.

GREIG, N.H., SWEENEY, D.J. & RAPOPORT, S.I. (1988). Comparative brain and plasma pharmacokinetics and anticancer activities of chlorambucil and melphalan in the rat. *Cancer Chemother. Pharmacol.*, **21**, 1.

HAFSTRÖM, L., RUDENSTAM, C.-M., HOLMBERG, S.B., SCHERSTÉN, T. & EHRSSON, H. (1990). The pharmacokinetics of melphalan in regional hyperthermic liver perfusion. *Reg. Cancer Treat.*, **3**, 23.

HOHN, D.C., STAGG, R.J., FRIEDMAN, M.A. & 5 others (1989). A randomized trial of continuous intravenous versus hepatic intra-arterial floxuridine in patients with colorectal cancer metastatic to the liver: the Northern California Oncology Group Trial. *J. Clin. Oncol.*, **7**, 1646.

HUGHES, K.S., SIMON, R., SONGHORABODI, S. & 46 others (1986). Resection of the liver for colorectal carcinoma metastases: a multi-institutional study of patterns of recurrence. *Surgery*, **100**, 278.

KEMENY, N. & GOLBEY, R. (1980). A chemotherapeutic approach to colorectal carcinoma. In *Neoplasms of the Colon, Rectum and Anus*, Stearns, M.R. Jr. (ed.), p. 155. John Wiley and Sons, New York.

KEMENY, N., DALY, J., REICHMAN, B., GELLER, N., BOTET, J. & ODERMAN, P. (1987). Intrahepatic or systemic infusion of fluorodeoxyuridine in patients with liver metastases from colorectal carcinoma. *Ann. Intern, Med.*, **107**, 459.

KEMENY, N., NEIDZWIECKI, D., SHURGOT, B. & ODERMANN, P. (1989). Prognostic variables in patients with hepatic metastases from colorectal cancer. *Cancer*, **63**, 742.

LAZARUS, H.M., GOTTFRIED, M.R., HERZIG, R.H. & 8 others (1982). Veno-occlusive disease of the liver after high-dose mitomycin C therapy and autologous bone marrow transplantation. *Cancer*, **49**, 1789.

LAZARUS, H.M., HERZIG, R.H., GRAHAM-POLE, J. & 9 others (1983). Intensive melphalan chemotherapy and cryopreserved autologous bone marrow transplantation for the treatment of refractory cancer. *J. Clin. Oncol.*, **1**, 359.

LEFF, R.S., THOMPSON, J.M., JOHNSON, D.B. & 5 others (1986). Phase II trial of high-dose melphalan and autologous bone marrow transplantation for metastatic colon carcinoma. *J. Clin. Oncol.*, **4**, 1586.

LOOS, U., MUSCH, E., ENGEL, M., HARTLAPP, J.H., HÜGL, E. & DENGLER, H.J. (1988). The pharmacokinetics of melphalan during intermittent therapy of multiple myeloma. *Eur. J. Clin. Pharmacol.*, **35**, 187.

MARINELLI, A., VAN DE VELDE, C.J.H., KUPPEN, P.J.K., FRANKEN, H.C.M., SOUVERIJN, J.H.M. & EGGERMONT, A.M.M. (1990a). A comparative study of isolated liver perfusion versus hepatic artery infusion with mitomycin C in rats. *Br. J. Cancer*, **62**, 893.

MARINELLI, A., PONS, D.H.A., KUPPEN, P.J.K., VREEKEN, J.A.C., TJADEN, U.R. & VAN DE VELDE, C.J.H. (1990*b*). Isolated liver perfusion (ILP) versus hepatic artery infusion (HAI) with 5-fluorouracil (FUra) and mitomycin C (MMC) in a rat model. *Proc. Am. Ass. Cancer Res.*, **31**, 429, 2547.

MARINELLI, A., DIJKSTRA, F.R., DIERENDONCK, J.H., KUPPEN, P.J.K., CORNELISSE, C.J. & VAN DE VELDE, C.J.H. (1991). Effectiveness of isolated rat liver perfusion with mitomycin C in the treatment of liver tumours of rat colorectal cancer. *Br. J. Cancer* **64**, 74.

MARQUET, R.L., WESTBROEK, D.L. & JEEKEL, J. (1984). Interferon treatment of a transplantable rat colon adenocarcinoma: importance of tumor site. *Int. J. Cancer*, **33**, 689.

MARTIN, J.K. Jr, O'CONNELL, M.J., WIENAND, H.S. & 6 others (1990). Intra-arterial floxuridine vs systemic fluorouracil for hepatic metastases from colorectal cancer. A randomized trial. *Arch. Surg.*, **125**, 1022.

MILLAR, B.C., TILBY, M.J., ORMEROD, M.G., PAYNE, A.W.R., JINKS, S. & LOVEROCK, P.S. (1986). Comparative studies of total cross-linking, cell survival and cell cycle perturbations in Chinese hamster cells treated with alkylating agents *in vitro*. *Biochem. Pharmacol.*, **35**, 1163.

MINOR, D.R., ALLEN, R.E., ALBERTS, D., PENG, Y.-M., TARDELLI, G. & HUTCHINSON, J. (1985). A clinical and pharmacokinetic study of isolated limb perfusion with heat and melphalan for melanoma. *Cancer*, **55**, 2638.

RADNELL, M., JEPPSSON, B. & BENGMARK, S. (1990). A technique for isolated liver perfusion in the rat with survival and results of cytotoxic drug perfusion on liver tumour growth. *J. Surg. Res.*, **49**, 394.

SILVERBERG, E. & LUBERA, J. (1988). *Cancer Statistics*. CA, **38**, 5.

SKIBBA, J.L., ALMAGRO, U.A., CONDON, R.E. & PETROFF, R.J. (1983). A technique of isolation perfusion of the canine liver with survival. *J. Surg. Res.*, **34**, 123.

SKIBBA, J.L., QUEBBEMAN, E.J., KOMOROWSKI, R.A. & THORSEN, K.M. (1988). Clinical results of hyperthermic liver perfusion for cancer in the liver. *Contr. Oncol.*, **29**, 222.

TAHA, I.A.-K., AHMAD, R.A., ROGERS, D.W., PRITCHARD, J. & ROGERS, H.J. (1983). Pharmacokinetics of melphalan in children following high-dose intravenous injection. *Cancer Chemother. Pharmacol.*, **10**, 212.

VAN DE VELDE, C.J.H., KOTHUIS, B.J.L., BARENBRUG, H.W.M. & 4 others (1986). A successful technique of in vivo isolated liver perfusion in pigs. *J. Surg. Res.*, **41**, 593.

VISTICA, D.T. (1979). Cytotoxicity as an indicator for transport mechanism. Evidence that melphalan is transported by two leucine-preferring carrier systems in the L1210 murine leukemia cell. *Biochem. Biophys. Acta*, **550**, 309.

VISTICA, D.T. (1983). Cellular pharmacokinetics of the phenylalanine mustards. *Pharmacol. Ther.*, **22**, 379.

WAGNER, J.S., ADSON, M.A., VAN HEERDEN, I.A., ADSON, M.H. & JESTRUP, D.M. (1984). The natural history of hepatic metastases from colorectal cancer. *Ann. Surg.*, **199**, 502.

WOOD, C.B., GILLIS, C.R. & BLUMGART, L.H. (1976). A retrospective study of the natural history of patients with liver metastases from colorectal cancer. *Clin. Oncol.*, **2**, 285.

ZWELLING, L.A., FILIPSKI, J. & KOHN, K.W. (1979). Effect of thiourea on survival and DNA cross-link formation in cells treated with platinum(II) complexes, L-phenylalanine mustard, and bis(2-chloroethyl)methylamine. *Cancer Res.*, **39**, 4989.